# Supplementary material for: Global comparative structural analysis of responses to protein phosphorylation
Source: Nat Commun. 2025 Oct 24;16:9407. doi: 10.1038/s41467-025-64116-4 (PMC12552441; doi:10.1038/s41467-025-64116-4)
Supplement: Supplementary file 1 — Supplementary Information [file 41467_2025_64116_MOESM1_ESM.pdf]

Supplementary information: Global comparative structural analysis of responses to protein phosphorylation

Miguel Correa Marrero[1,2,3], Victor Hugo Mello[4], Pablo Sartori[4], and Pedro Beltrao*[1,2,3,4]

[1]European Molecular Biology Laboratory, European Bioinformatics Institute (EMBL-EBI), Wellcome Genome Campus, Hinxton, CB10 1SD, United Kingdom
[2]Institute of Molecular Systems Biology, Department of Biology, ETH Zurich, Otto-Stern-Weg 3, Zurich, 8093, Switzerland
[3]SIB Swiss Institute of Bioinformatics, Lausanne, Switzerland
[4]Gulbenkian Institute for Molecular Medicine, Lisbon, Portugal

1 Supplementary figures

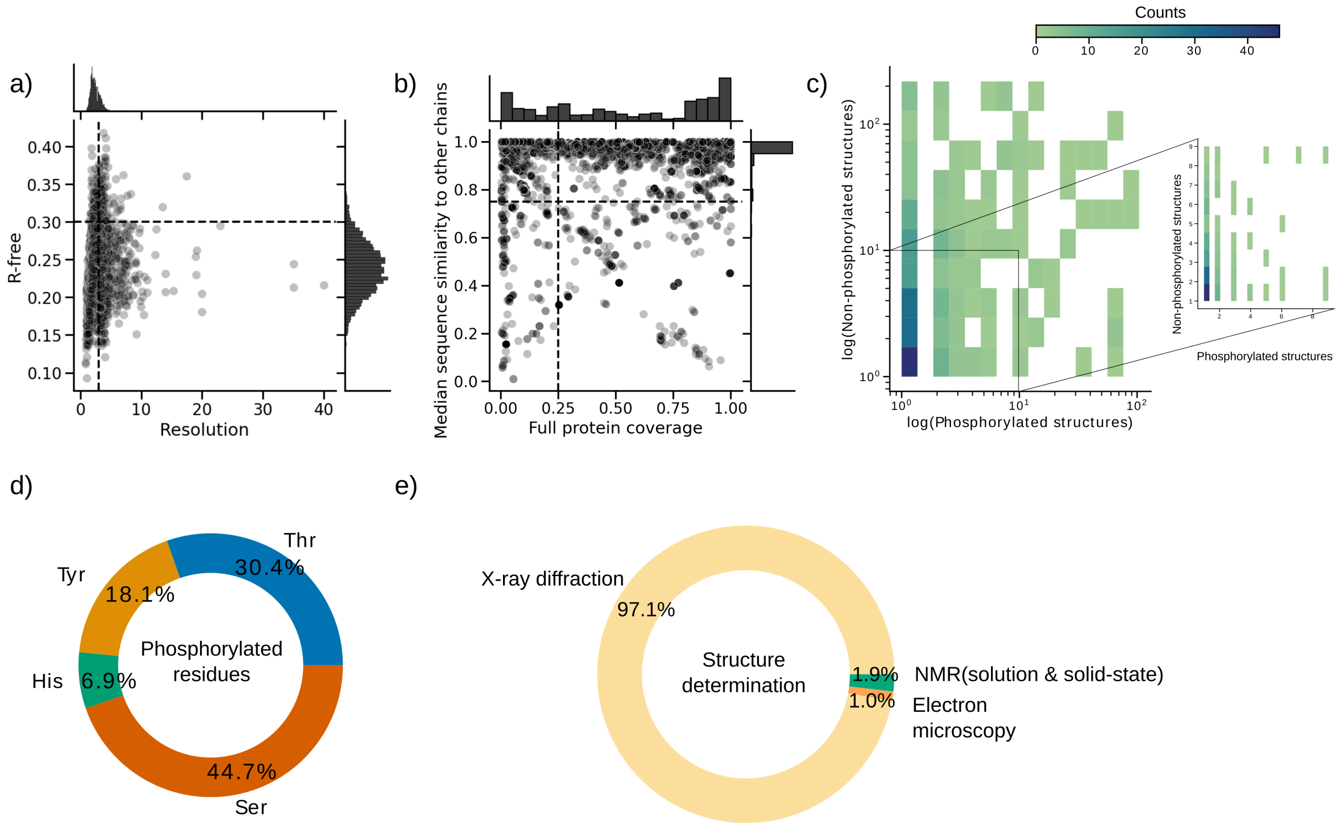

Supplementary Figure 1: Curation of structural data. a) Scatterplot of structural resolution versus R-free for each retrieved structure. Dashed lines indicate selected cutoff points. b) Scatterplot of structural coverage (relative to the full UniProt canonical sequence) versus the median sequence similarity to all other structures of the same protein in the dataset. The latter serves as a metric of overlap. Dashed lines indicate selected cutoff points. c) 2D histogram showing the number of phosphorylated structures versus the number of non-phosphorylated structures for each protein in the filtered dataset. The inset focuses on the range from zero to 10 structures. d) Donut chart displaying the distribution of different phosphorylated residues in the filtered dataset. e) Donut chart illustrating the methodologies used to solve the structures in the filtered dataset. Source data are provided as a Source Data file.

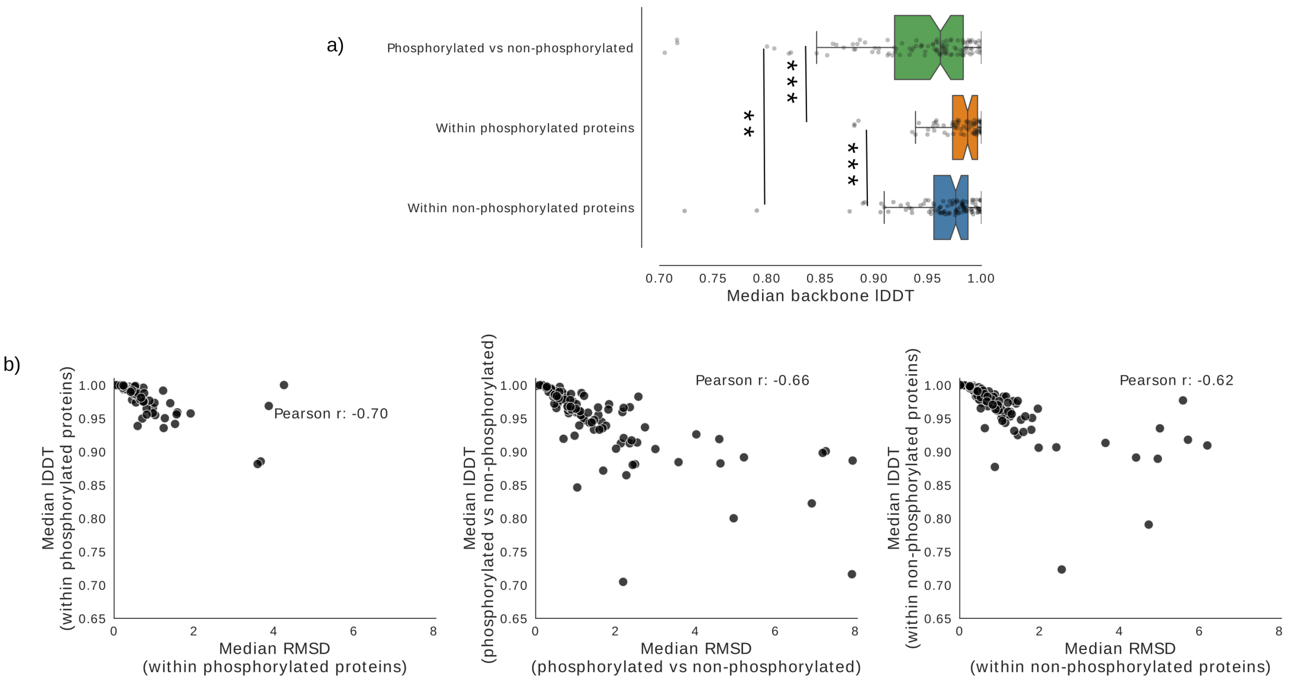

Supplementary Figure 2: Effects of phosphorylation on protein backbone conformation, as measured by the Local Distance Difference Test (lDDT), a superposition-free structural similarity metric, on a random subset of 30% of the phosphosites in the dataset. The lDDT score ranges from 0 to 1, where 0 is the lowest possible similarity and 1 the highest. a) Boxplot of conformational changes between phosphorylated and non-phosphorylated structures, and structural variability within each set. Points represent median changes per phosphosites. Notches indicates 95% confidence intervals. Outliers below 0.7 not shown. As in the main text, differences between the distribution of median lDDTs between phosphorylated and non-phosphorylated structures ($n = 110$) and within group comparison distributions ($n = 96$ for the comparison within non-phosphorylated structures, $n = 72$ for the comparison within phosphorylated structures; we use only cases where at least two structures were available per comparison) were assessed with one-tailed Mann-Whitney U tests, finding significant differences in both cases (phosphorylated vs. non-phosphorylated against within non-phosphorylated proteins, p-value $= 9.1 \times 10^{-3}$; phosphorylated vs. non-phosphorylated against within phosphorylated proteins, p-value $= 2.6 \times 10^{-6}$). The comparison of the distribution between the two within group comparisons was carried out with a one-sided Wilcoxon rank sum test (p-value $= 1.0 \times 10^{-3}$). b) Scatterplots showing the correlation between RMSDs and lDDT scores in the different sets. Source data are provided as a Source Data file.

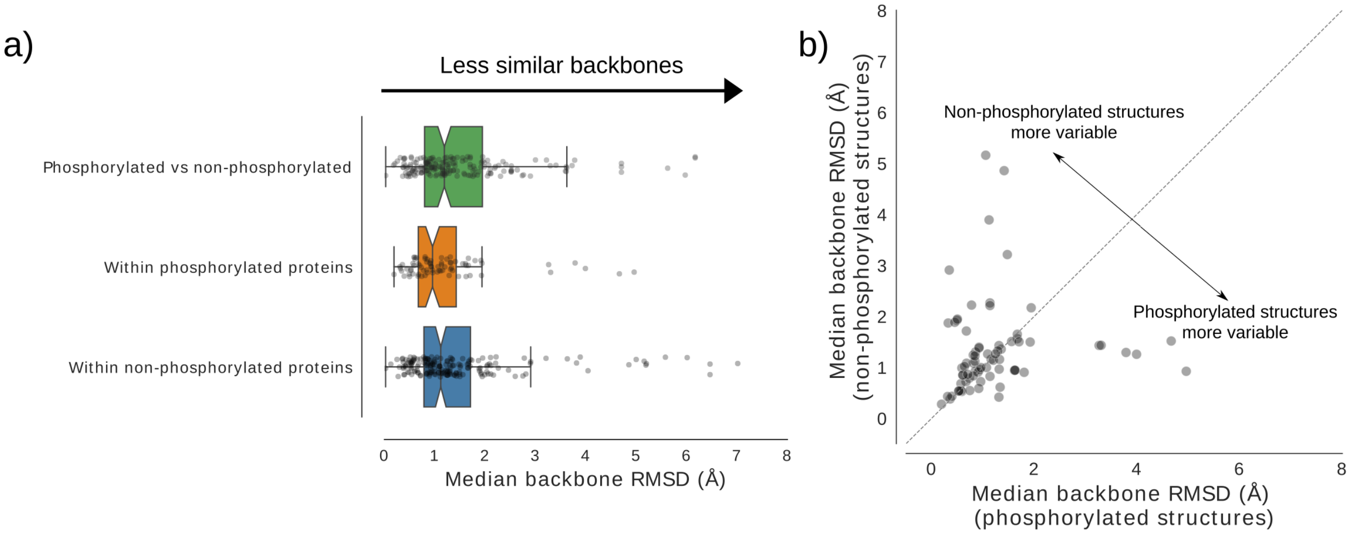

c)

Median backbone RMSD	Excluding comparisons between structures with the same lead author: No	Yes
Phosphorylated vs non-phosphorylated	1.14 ± 3.13	1.19 ± 2.29
Within phosphorylated proteins	0.65 ± 1.12	0.95 ± 1.24
Within non-phosphorylated proteins	0.89 ± 1.59	1.12 ± 1.83

Supplementary Figure 3: Investigating the influence of the lead author effect on observed conformational changes. To control for possible batch effects due to structures being produced by the same research lab, we excluded all pairwise comparisons of structures with the same lead author. a) Boxplot of conformational changes between phosphorylated and non-phosphorylated structures, and structural variability within each set, after excluding comparisons between structures sharing the same lead author ($n = 78$ phosphosites). Points represent median changes per phosphosite. Notches indicate 95% confidence intervals. b) Scatterplot comparing median backbone RMSD per phosphosite within phosphorylated structures (x-axis) to non-phosphorylated counterparts (y-axis). The diagonal line represents the identity line; 59% of points are above the line. Only includes phosphosites with at least two structures in each state. After excluding comparisons betweeen structures sharing the same lead author, 78 such phosphosites are available. c) Table showing the median backbone RMSD for different comparisons over the whole dataset, either before excluding comparisons between structures sharing the same lead author (left) or after (right). Source data are provided as a Source Data file.

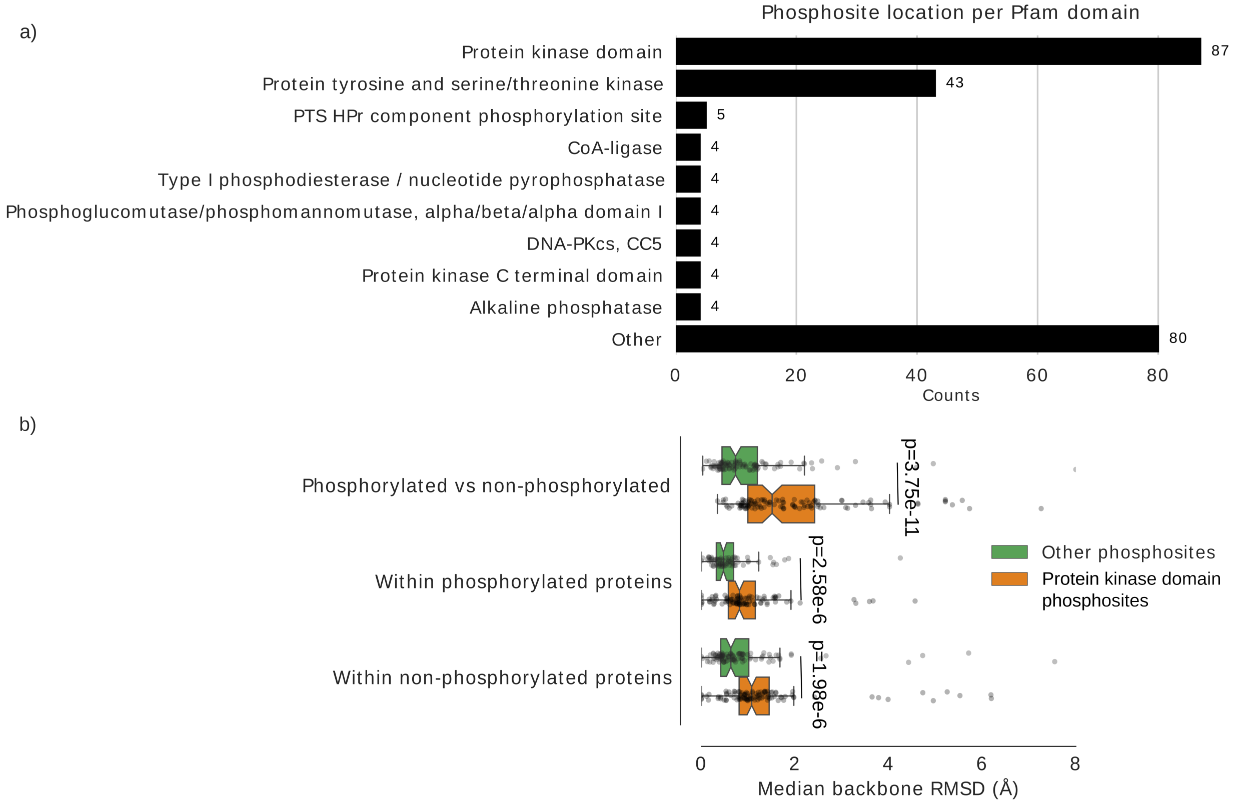

Supplementary Figure 4: Protein family over-representation and its impact on observed conformational changes. a) Bar plot showing the number of phosphosites per Pfam domain identified in our dataset. A clear enrichment of phosphosites within protein kinase domains is observed. Note that not all phosphosites in our dataset are within Pfam domains; as such, they are not counted in this plot. b) Boxplot of conformational changes between phosphorylated and non-phosphorylated structures, and structural variability within each set, according to whether the phosphosite is located within a protein kinase domain (orange) or not (green). Sample sizes are as follows: phosphorylated vs non-phosphorylated comparison — kinase, $n = 115$; non-kinase, $n = 105$; within the non-phosphorylated set — kinase, $n = 107$; non-kinase, $n = 100$; within the phosphorylated set — kinase, $n = 97$; non-kinase, $n = 73$. Points represent median changes per phosphosite. Notches indicate 95% confidence intervals; outliers beyond 8 Å not shown. Source data are provided as a Source Data file.

a)

	Coefficient	p-value
Intercept	1.42	<0.001
Number of unique protein partners (phosphorylated)	0.18	<0.001
Number of unique protein partners (non-phosphorylated)	0.01	0.442
Number of shared protein partners	-0.14	0.05

b)

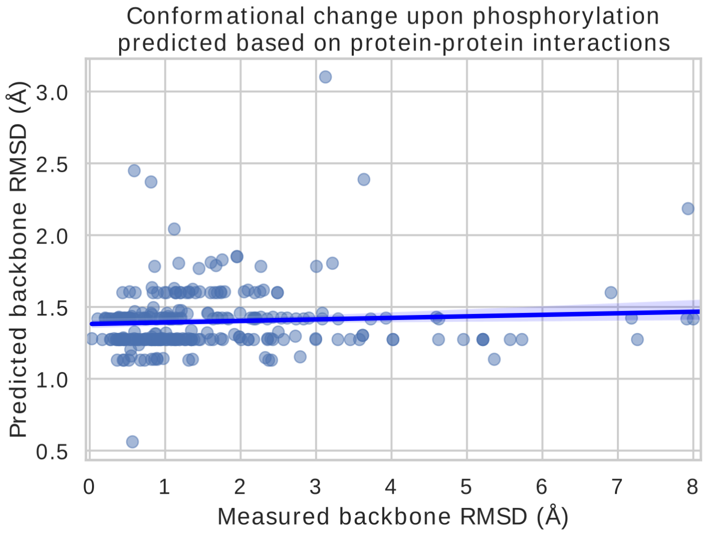

Supplementary Figure 5: Investigating the effect of protein-protein interactions on observed conformational changes upon phosphorylation. a) Summary of a robust linear regression model describing the relationship between the number of protein-protein interactions and observed backbone conformational change upon phosphorylation. b) Scatterplot of observed versus predicted conformational changes (n = 290 phosphosites). The predictions are based on the aforementioned robust linear model based on protein-protein interactions. The shaded band represents the 95% confidence interval for the regression line. Data points with observed RMSD values greater than 8 Å on the x-axis are excluded for clarity. Source data are provided as a Source Data file.

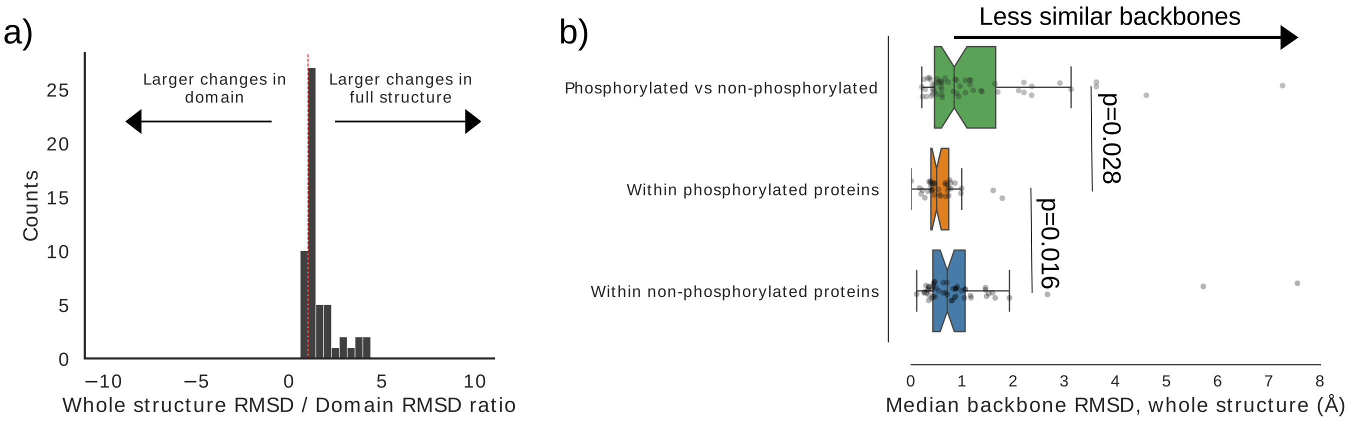

Supplementary Figure 6: Comparison of the effect of phosphorylation on protein backbones in protein domains versus the whole structure. a) Histogram of the ratio of backbone RMSD in the whole structure to backbone RMSD only in the phosphorylated Pfam domain. A ratio greater than 1 indicates greater changes in the overall structure than in the phosphorylated domain. b) Boxplot of conformational changes between phosphorylated and non-phosphorylated structures (using the whole structure), and structural variability within each set. As in the main text, differences between the distribution of median RMSDs between phosphorylated and non-phosphorylated structures (n = 57 phosphosites) and within group comparison distributions (n = 52 for the comparison within non-phosphorylated structures, n = 37 for the comparison within phosphorylated structures; we use only cases where at least two structures were available per comparison) were assessed with one-tailed Mann-Whitney U tests). Points represent median changes per phosphosite. Notches indicate 95% confidence intervals; outliers beyond 8 Å not shown. Source data are provided as a Source Data file.

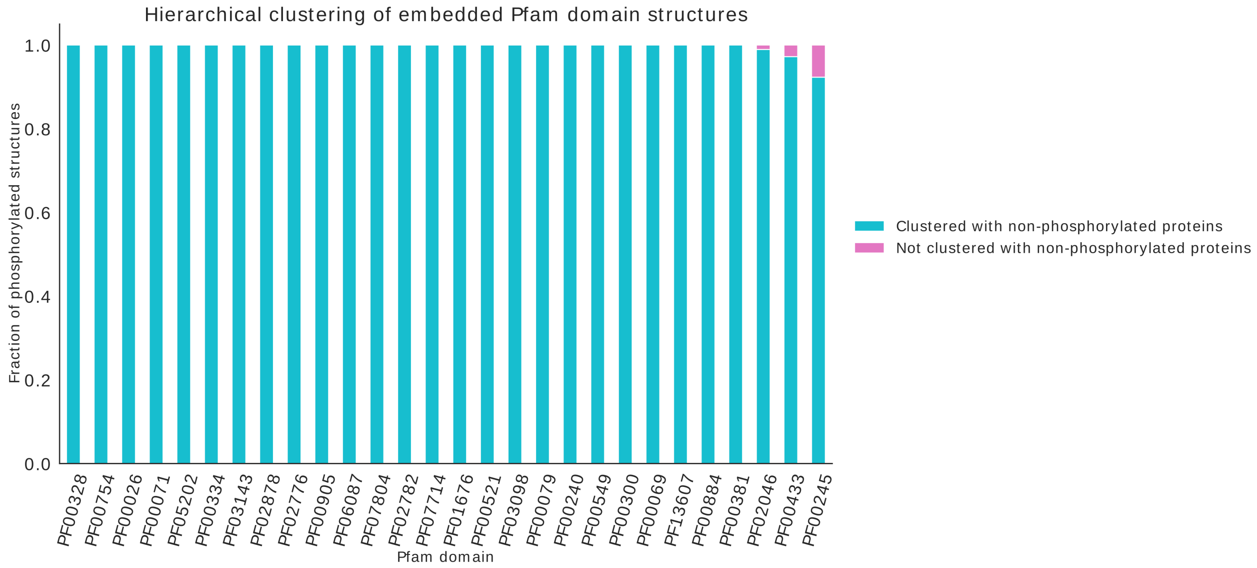

Supplementary Figure 7: Stacked bar chart summarizing the proportion of phosphorylated structures, per domain, that are within a cluster containing at least one non-phosphorylated structure. In this analysis, clustering was performed directly in the high-dimensional embedding space using agglomerative hierarchical clustering with cosine distance and average linkage, rather than using HDBSCAN on the PCA-reduced representations as in the main text. Note that, unlike HDBSCAN, hierarchical clustering assigns all samples to a cluster. Source data are provided as a Source Data file.

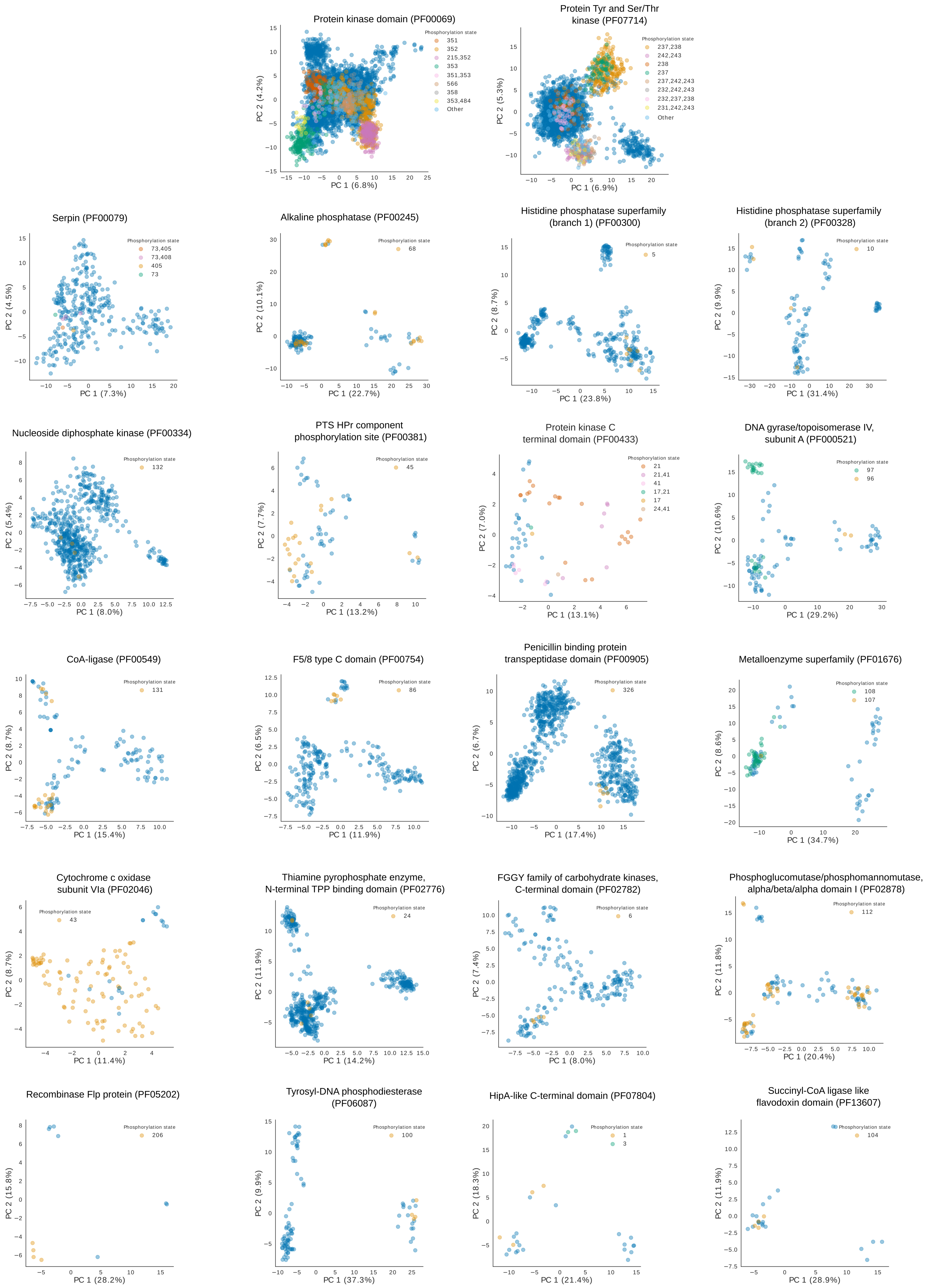

Supplementary Figure 8: Additional examples of low-dimensional, domain-specific conformational landscapes. Blue dots represent non-phosphorylated structures, while other colors indicate specific phosphorylation states. For clarity, due to the high number of different phosphorylation states observed in the protein kinase domain (PF00069) and protein tyrosine and serine/threonine kinase (PF07714; both displayed on top), only the 8 most common states (besides non-phosphorylated proteins) are colored distinctly. Phosphosite indexes in the legend are assigned based on sequence alignments of the analyzed structures. Source data are provided as a Source Data file.

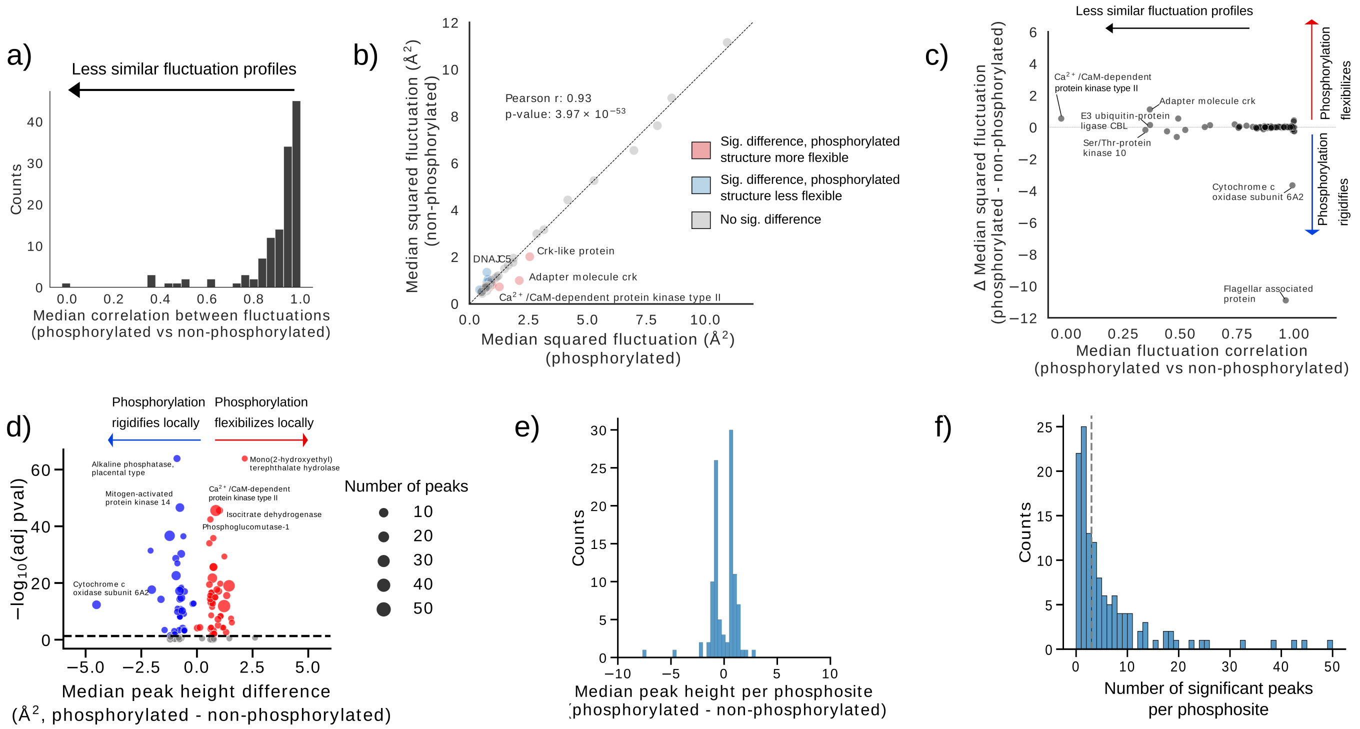

Supplementary Figure 9: Changes in predicted global and local residue fluctuations. a) Histogram showing the median correlation in residue fluctuation profiles between phosphorylated and non-phosphorylated structures. b) Scatterplot comparing the median fluctuations of phosphorylated structures (x-axis) to their non-phosphorylated counterparts. Among cases with significant changes, ~47% showed increased and ~53% showed decreased flexibility. DNAJC5 refers to DnaJ homolog subfamily C member 5. Outliers beyond 12 Å are excluded. The p-value of the Pearson correlation ($n = 128$) is computed using a two-sided test. c) Scatterplot comparing the median correlation in residue fluctuations between phosphorylated and non-phosphorylated structures (x-axis) with the difference in median protein-wise fluctuation between phosphorylated and non-phosphorylated proteins (y-axis). d) Scatterplot illustrating the magnitude and significance of local changes in dynamics. Bubble size proportional to the number of significant local peaks identified. e) Histogram of the number of significant local peaks (i.e., differences in residue fluctuations exceeding a specified threshold) per phosphosite. The dashed line indicates the median number of peaks (three). f) Histogram of the median height of each peak per phosphosite. Overall negative values indicate greater fluctuations in the non-phosphorylated protein; overall positive values, the reverse. Source data are provided as a Source Data file.

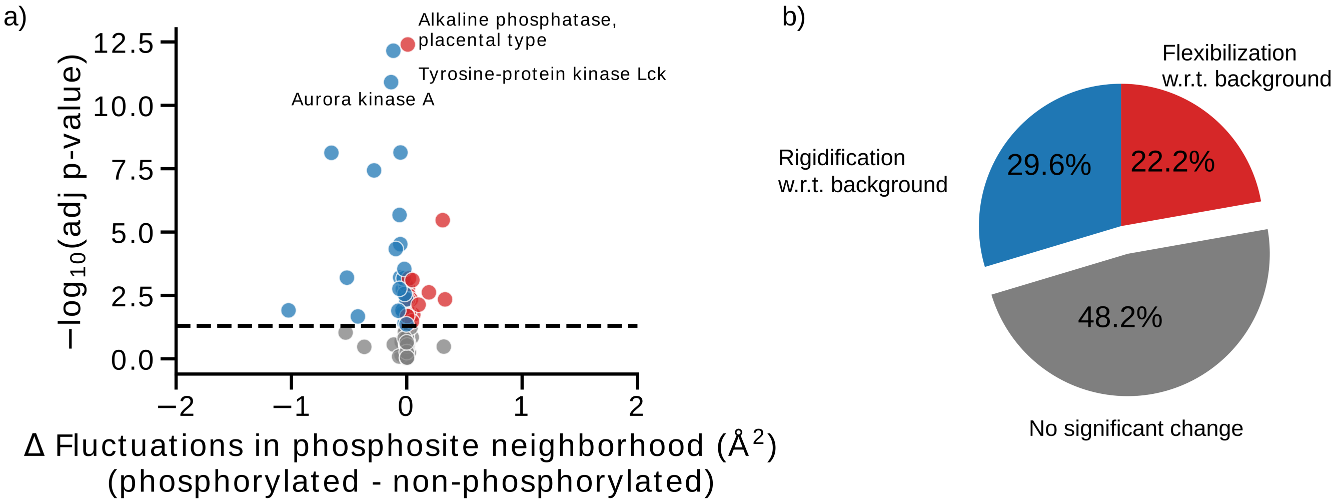

Supplementary Figure 10: Local changes in predicted dynamics in the neighborhood of the phosphosite with respect to the rest of the protein. 51.8% of phosphorylation events are associated with such changes, which are typically extremely minor (median change with respect to background: -7.6×10^{-3} Å^2, among significant cases) and with no consistent direction of the effect. a) Scatterplot illustrating the magnitude and significance of changes in dynamics in the phosphosite neighborhood with respect to the background upon phosphorylation ($n = 108$). b) Pie chart categorizing the types of local changes in dynamics in the phosphosite neighborhood. Source data are provided as a Source Data file.

a)

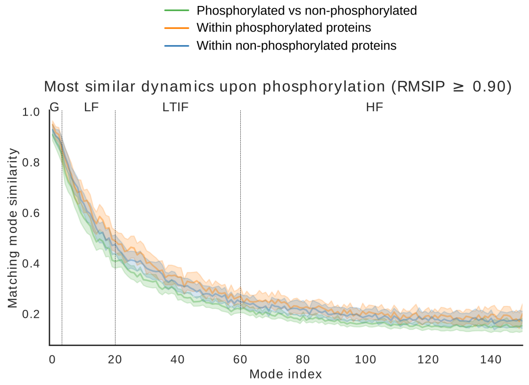

N	100
Adj. R squared	0.419
F-statistic	12560
p-value	<0.001

	**Coefficient**	**p-value**
Intercept	0.636	<0.001
Comparison between groups	-0.0299	<0.001
Comparison within phosphorylated	0.016	<0.001
sqrt(Mode index)	-0.0395	<0.001

b)

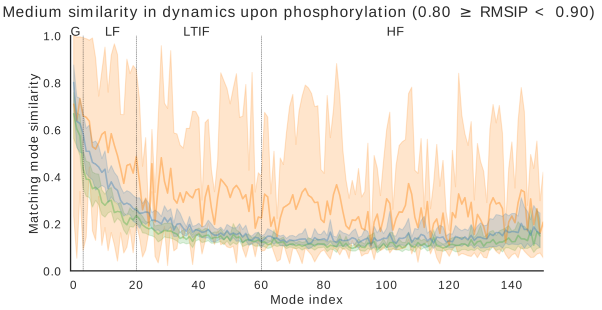

N	18
Adj. R squared	0.259
F-statistic	679.8
p-value	<0.001

	**Coefficient**	**p-value**
Intercept	0.371	<0.001
Comparison between groups	-0.035	<0.001
Comparison within phosphorylated	0.192	<0.001
sqrt(Mode index)	-0.022	<0.001

c)

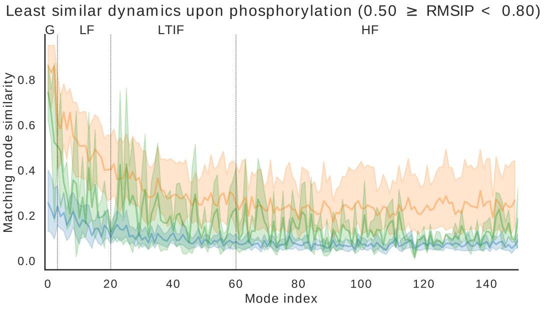

N	10
Adj. R squared	0.195
F-statistic	358.8
p-value	<0.001

	**Coefficient**	**p-value**
Intercept	0.252	<0.001
Comparison between groups	-0.053	<0.001
Comparison within phosphorylated	0.111	<0.001
sqrt(Mode index)	-0.008	<0.001

Supplementary Figure 11: Comparison of normal modes in different bins of dynamics similarity, from most similar (top) to least similar (bottom). Left column, similarity of matching pairs of modes over different frequency regimes. Bands indicate 95% confidence intervals. Right column, summary of linear regressions modelling the relationship between mode similarity for each bin. All regressions were statistically significant, as were the model coefficients, indicating significant differences in normal modes regardless of the bin. Source data are provided as a Source Data file.

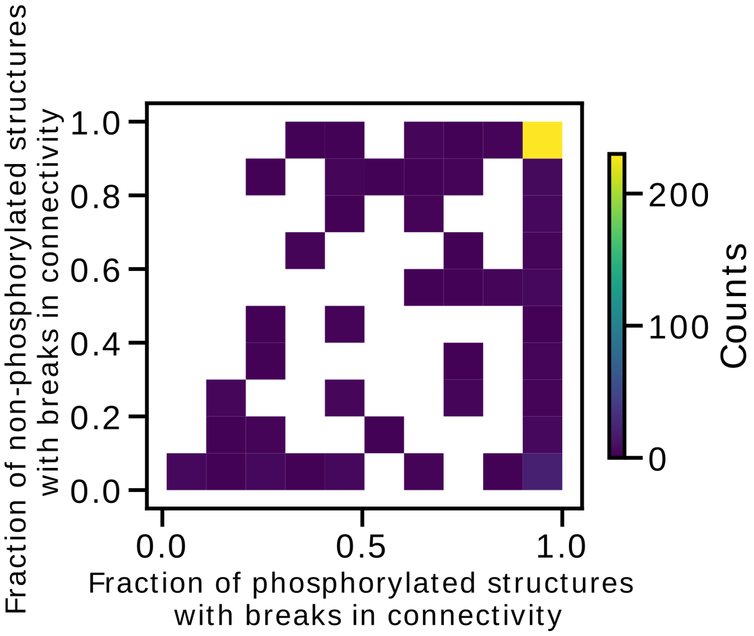

Supplementary Figure 12: 2D histogram showing the number of phosphorylated and non-phosphorylated structures that were filtered out (for all 347 unique phosphosites) for normal mode analysis due to breaks in connectivity. Source data are provided as a Source Data file.

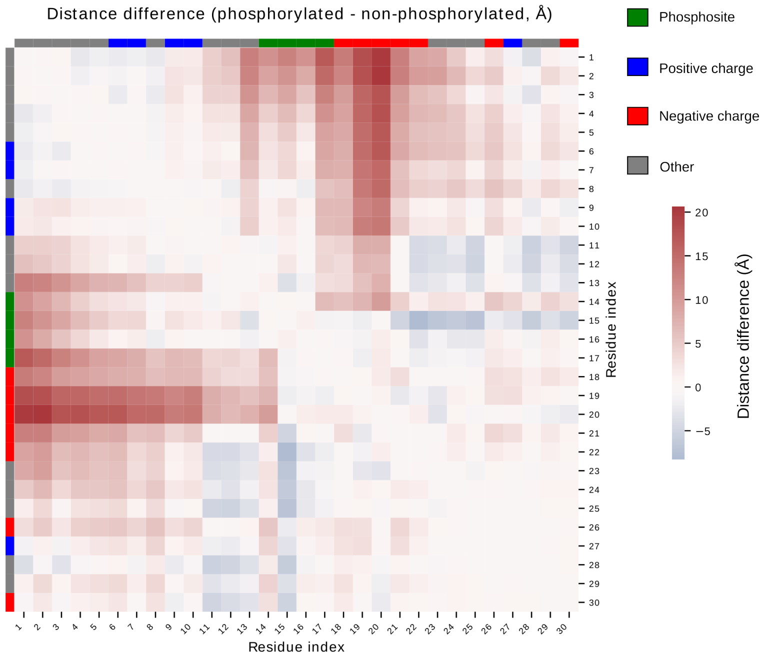

Supplementary Figure 13: Heatmap of pairwise residue distance differences between phosphorylated and non-phosphorylated states for the chromobox protein homolog 5 example, showing a window centered around the phosphosites in the disordered region. Each cell shows the change in Cβ–Cβ distance (phosphorylated minus non-phosphorylated, in Å) between residue pairs. Red indicates an increase in distance upon phosphorylation; blue indicates a decrease. Colors along the axes denote residue properties: phosphosites (green), positively charged (blue), negatively charged (red), and others (gray). Source data are provided as a Source Data file.

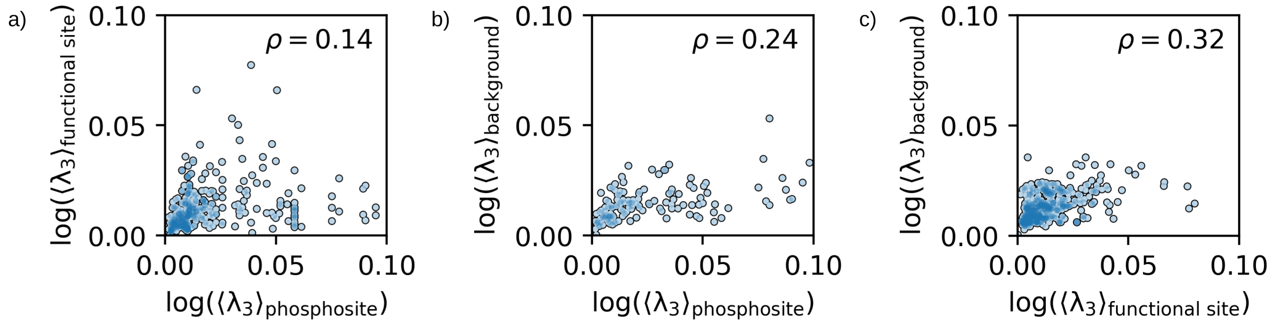

Supplementary Figure 14: Correlation analysis of the mean extensive stretches between three groups: phosphosites ($\pm$ 2 residues), functional sites, and background residues. Although the observed correlations were consistently relatively low, they were nonetheless statistically significant compared to the null hypothesis of uncorrelated, normally distributed data ($p = 0.001$, $p = 0.003$, and $p = 6 \times 10^{-12}$, respectively). Sample sizes were $n = 438$ site pairs for panel a, $n = 179$ for panel b, and $n = 438$ for panel c. Source data are provided as a Source Data file.

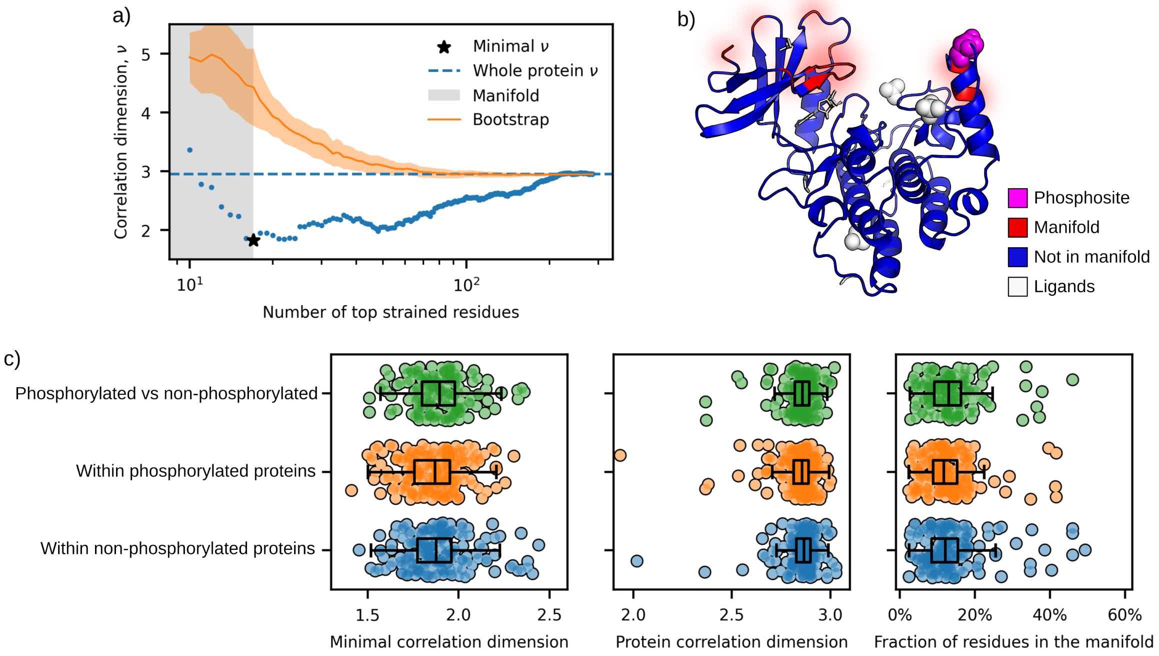

Supplementary Figure 15: Strain manifold analysis reveals a conserved structural architecture between phosphorylated and non-phosphorylated proteins. a) Example of manifold determination using the correlation dimension ν for residues ranked by strain (PDBs 7P7F_B vs. 4HNF_A). The correlation dimension values are plotted as a function of the number of top-strained residues, with the minimum (denoted by a star) indicating the residues belonging to the high-strain manifold. Bootstrapping (mean and standard deviation, $n = 40$) demonstrates that randomly distributed strained residues do not form a low-dimensional manifold. b) Spatial visualization of residues within the strain manifold, where strain is concentrated in the upper region of the structure. c) General properties of high-strain manifolds remain consistent across comparisons between different groups, with no significant differences ($p > 0.05$, Kruskal-Wallis test) in manifold dimension, overall protein correlation dimension, or the proportion of residues within the manifold. Sample sizes were $n = 157$ phosphosites in the comparison between phosphorylated and non-phosphorylate structures, $n = 202$ in the comparison within phosphorylated proteins, and $n = 176$ in the comparison within non-phosphorylated proteins. Source data are provided as a Source Data file.

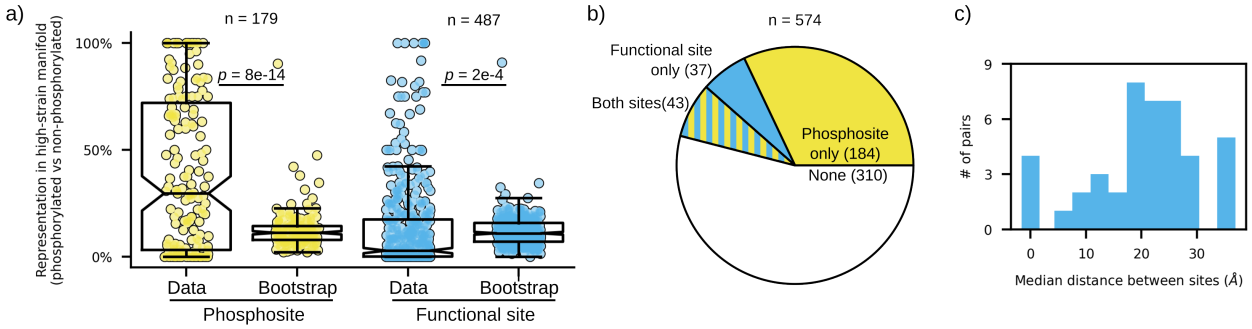

Supplementary Figure 16: Representation of phosphosites and functional sites in the high-strain manifold. a) Boxplot of the representation of phosphosites and functional sites in the manifold, averaged across all pairwise comparisons per phosphosite. Bootstraps were generated by randomising the strain distribution over different residues ($n = 50$). Statistical significance was determined using two-tailed Wilcoxon signed-rank tests. b) Share of pairs of phosphosite / functional sites with high representation in the manifold. We identify each site as significantly represented by comparing them individually to the bootstraps of the same protein when satisfying the following criterion: $\langle R \rangle^{\text{data}} > \frac{1}{n} \sum_i^n \langle R \rangle_i^{\text{bootstrap}} + 3\sigma^{\text{bootstrap}}$, where σ is the standard deviation of the representation in the bootstrap samples. c) Histogram of distances between phosphosite and functional site pairs identified to belong to the high-strain manifold. Source data are provided as a Source Data file.
