# Supplementary material for: Global comparative structural analysis of responses to protein phosphorylation
Source: Nat Commun. 2025 Oct 24;16:9407. doi: 10.1038/s41467-025-64116-4 (PMC12552441; doi:10.1038/s41467-025-64116-4)
Supplement: Supplementary file 2 — Description of Additional Supplementary Files [file 41467_2025_64116_MOESM2_ESM.pdf]

File Name: Supplementary Data 1
Description: Curated dataset of paired phosphorylated and non-phosphorylated protein structures.

File Name: Supplementary Data 2
Description: Table of obtained measurements per phosphosite.

File Name: Supplementary Data 3
Description: Table of phosphosites with evidence of mechanical coupling to known functional sites.
